# Complementary and Alternative Medicine Use among Norwegian Cancer Survivors: Gender-Specific Prevalence and Associations for Use

**DOI:** 10.1155/2013/318781

**Published:** 2013-03-28

**Authors:** Agnete E. Kristoffersen, Arne J. Norheim, Vinjar M. Fønnebø

**Affiliations:** Department of Community Medicine, National Research Center in Complementary and Alternative Medicine (NAFKAM), University of Tromsø, N-9037 Tromsø, Norway

## Abstract

The associations for CAM use are only occasionally differentiated by gender in populations where both male and female cancer survivors occur. The aim of this study is to describe the prevalence of CAM use in individuals with a previous cancer diagnosis and to investigate gender differences regard to factors associated with use. A total of 12982 men and women filled in a questionnaire with questions about life style and health issues. Eight hundred of those had a previous cancer diagnosis of whom 630 answered three questions concerning CAM use in the last 12 months. A total of 33.8% of all cancer survivors reported CAM use, 39.4% of the women and 27.9% of the men (*P* < 0.01). The relationship between the demographic variables and being a CAM user differed significantly between men and women with regard to age (*P* = 0.03), education (*P* = 0.04), and income (*P* < 0.01). Female CAM users were more likely to have a university degree than the nonusers, while male CAM users were more likely to have a lower income than the nonusers. According to this study, prevalence and factors associated with CAM use differ significantly between male and female survivors of cancer.

## 1. Introduction

Although self-reported use of complementary and alternative medicine (CAM) among cancer patients is increasing [1–4], studies report substantial difference in the level of use ranging from 7% [5] to 95% [6]. This wide range in self-reported use could be due to differences in the definition of a CAM user [7, 8] and/or differences in the time frame of the use [9].

Younger, highly educated women have been described as the most frequent users of CAM [4, 8, 10–12]. Frequent use has also been reported among patients with symptoms related to their cancer, patients receiving only palliative treatment, patients with metastatic disease, and patients diagnosed with cancer more than three months previously [13].

Others again report that use of, or interest in, CAM is predicted by younger age, progressive cancer, and active coping behaviour [14]. CAM use related to time after diagnosis has also been studied [9]. Likelihood of death occurring from the cancer has been reported to be both associated [15, 16] and not associated [17, 18] with CAM use. Likelihood of consulting a CAM provider has been associated with a university degree, low-perceived global health, and recent health complaints [19]. 

The predictors for CAM use in whole populations and among female cancer survivors have been described, while predictors for CAM use in male cancer survivors are still insufficiently studied in all cancer categories except prostate [20]. The reported reasons for CAM use have been only occasionally differentiated by gender in populations where both male and female cancer survivors occur [21, 22].

Since women with cancer are documented to use different kinds of CAM than men [21, 23] and that other patient groups are found to have gender-specific correlations for use [22, 24, 25], it is important also to investigate if the factors *associated* with CAM use in cancer are gender specific. 

The aim of this study is (1) to describe prevalence of CAM use in individuals with a previous cancer diagnosis and (2) to investigate whether men and women differ with regard to sociodemographical and health-related factors associated with CAM use.

## 2. Materials and Methods

The Tromsø Cohort Study series are a single-centred prospective and population-based health surveys of the adult inhabitants of the municipality of Tromsø, Northern Norway [26]. The population of Tromsø reflects the distribution of gender, educational level, and average income in Norway overall, but the population is somewhat younger [27]. The design includes repeated population health surveys to which total birth cohorts and random samples are invited. The Tromsø Cohort study collects information on a wide range of health-related issues, using questionnaires and health screenings. Use of CAM is collected through two different questionnaires.

**Figure 1:**
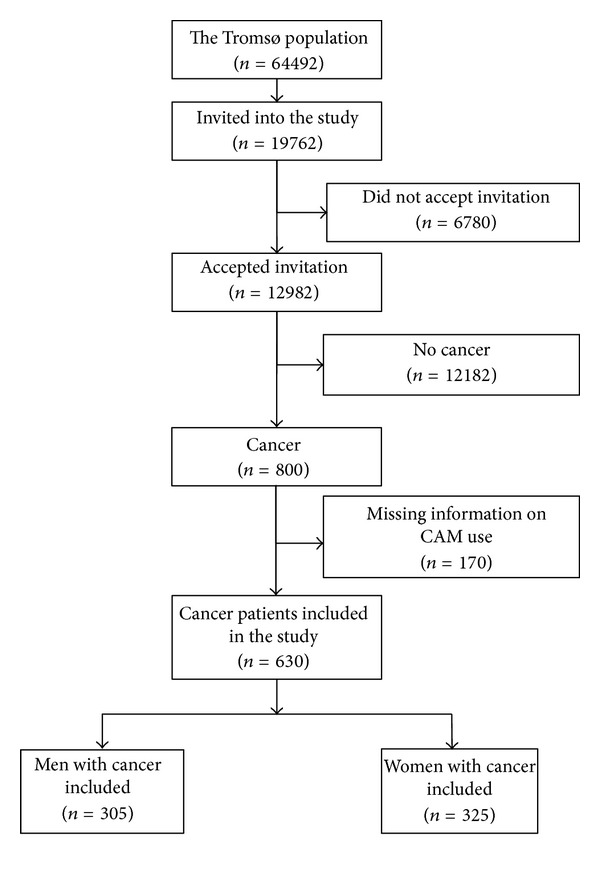
Flow chart that shows the selection of the studied population.

This paper is based on data from the sixth Tromsø study conducted in 2007/2008, including 12982 participants, 6053 men and 6929 women aged between 30 and 87 years old (response rate is 65.7%, 62.9% of the men and 68.4% of the women). Eight hundred of these participants have had cancer prior to the survey according to the Cancer Registry of Norway. Sixty-five men and 105 women failed to answer all the three questions concerning CAM use and were excluded from the analyses. This leaves us with 630 informants who responded to all three questions about CAM use, constituting the studied population (Figure 1). 

The letter of invitation contained a short questionnaire developed specifically for the sixth Tromsø study including use of a CAM provider. Individuals who attended the survey by answering the first questionnaire and undergoing a health screening, received subsequently a *second, more detailed, *questionnaire which they were asked to complete onsite or at home and return by mail. The questions concerning use of OTC products and self-techniques were placed in this *second* questionnaire.

The two questionnaires included questions on general state of health, diseases suffered by the respondent or their family, muscle pain and physical discomfort, food habits, alcohol consumption, smoking habits, physical activity in leisure time, level of education, use of medicine, and use of health services including CAM. The questions regarding CAM use were not related to any specific disease condition.

Study participants were classified as “CAM-users” by checking Yes for one or more of the three questions concerning visits to a CAM provider, use of CAM over-the-counter products (OTC), and CAM techniques (displayed in Table 2). Accordingly, a participant who checked No for all the three specific CAM-questions was classified as a nonuser. 

Informants who had seen a chiropractor were not defined as CAM users in this study as chiropractors are regulated health care personnel in Norway. This also applies to informants who had used cod liver oil, fish oil capsules, Omega-3, or ordinary vitamins/mineral supplements as these supplements are commonly used in the Norwegian population.

In Norway, an alternative medical provider is commonly understood as a practitioner providing CAM both as an alternative to and complementary to conventional treatment. A CAM provider offers therapies that are not commonly offered within the public health care service and are paid out-of-pocket by the patients themselves.

With a statistical power of 80% and using an alpha of 0.05, we were able to report a statistically significant within-gender differences in reported use of approximately 10 percentage points when cross tabulating use with other dichotomous variables. 

Associations for CAM use in men and women were analysed using chi-square tests in SPSS Windows (version 19.0, SPSS Inc., Chicago, IL), one variable at a time. Interaction between women and men concerning associations was investigated by testing homogeneity of the odds ratio in a multivariate analysis.

The data inspectorate has been notified about the study, and the regional ethics committee has recommended it. The participants have given their informed written consent.

## 3. Results

### 3.1. Basic Characteristics of the Studied Participants

The studied population (*n* = 630) consisted of 325 women and 305 men. Most cancer sites were represented, though breast cancer dominated among women (37.8%) and prostate cancer (34.8%) among men. Mean time since diagnosis was 10.6 years, 12 years in women and 9.4 years in men. Only 30 participants (ten women and 20 men) were less than 12 months after diagnosis. Most of the men (84.4%) and half of the women (56.8%) were living with a spouse/partner, and more than half of the participants reported good or excellent health (53%). Mean self-reported health was 73.7, ranging from 5 to 100 on a 100 point scale where 100 was the best imaginable health. Very few reported poor health (9%) despite a cancer diagnosis and a median age of 66 (Table 1).

### 3.2. Prevalence of CAM Use in the Cancer Patients

A total of 33.8% of all cancer survivors reported CAM use, 39.4% of the women and 27.9% of the men (*P* < 0.01). OTC products were most often used, used by 29% of the women and 20% of the men. A CAM provider was seen by 13% of the population, 16% of the women and 9% of the men. CAM techniques were least used, 7% of the women and only 2% of the men (Table 2). 

There were no significant differences in CAM use according to time since diagnosis and self-reported health, neither among men nor women.

Nonresponders could be included in the analysis by including informants answering “yes” to at least one of the three questions concerning CAM in the *CAM group* and all the patients with no or missing response to all the three questions were included in the *no CAM group. *The prevalence of CAM use would then have been 30.5% (*n* = 244), 35.3% among women (*n* = 152) and 24.9% among men (*n* = 92).

The cancer patients did not differ significantly from the group without cancer when the use of a CAM provider, CAM techniques, and OTC products were analysed separately. When the three CAM modalities were analysed together (CAM level 3 [23]), men with cancer were significantly more likely to be CAM users than men without cancer (27.9% versus 22.1%, *P* = 0.02). 

### 3.3. Factors Associated with CAM Use in Cancer Patients

There were no overall significant differences between users and nonusers of CAM in relation to age, education, income, self-reported health, time since diagnosis, or metastasis at first diagnosis. We found that CAM users were significantly more likely to be women (*P* = 0.002) and more likely to have breast cancer (*P* = 0.02).

The relationship between the demographic variables and being a CAM user differed significantly between men and women with regard to age (*P* = 0.03), education (*P* = 0.04), and income (*P* < 0.01) (Table 3). It was, therefore, necessary to present data stratified by gender.

When analysed separately, we found that university education (*P* < 0.01) and breast cancer (*P* < 0.01) was significantly associated with CAM use in women. We found no significant associations for age, income, or self-reported health in women (Tables 3 and 4). As breast cancer was significantly associated with CAM, the same analysis was conducted without breast cancer with the same result, however, no longer at a significant statistical level. 

When the three CAM modalities CAM provider (Table 5), OTC products (Table 6), and CAM techniques (Table 7) were analysed separately, we found that university education and younger age was associated with the use of CAM techniques and university education to be associated with the use of OTC products in women. 

Among men, we found that lower income was significantly associated with CAM use (*P* = 0.016). University education, age (Table 3), and self-reported health (Table 4) were not significantly associated with CAM use in men, though older age seemed to be a tendency (*P* = 0.072, Table 3). As prostate cancer was the most common cancer site among men, the same analyses was conducted without prostate cancer with the same result to ensure that the associations found were associated with men in general and not with prostate cancer in particular. 

When the three CAM modalities CAM provider (Table 5), OTC products (Table 6), and CAM techniques (Table 7) were analysed separately, we did not find age, income, or university education to be associated with use at a significant level. A tendency was, on the other hand, found for older age (*P* = 0.065) and lower family income (*P* = 0.085) in the use of OTC products in men.

When analysing interaction in CAM use between men and women, we found significant interactions in overall CAM use concerning age, university education, and family income (Table 3). We did not find significant interaction concerning the use of neither a CAM provider nor CAM techniques. In the use of OTC products, on the other hand, we found significant interactions concerning age and university education in men and women (Table 6).

## 4. Discussion

This study has shown that women were more likely to have used CAM than men and that the associations for CAM use differ between men and women. 

### 4.1. Bias

The cancer registry of Norway includes all patients diagnosed with cancer in Norway since 1952. This should ensure that the selected cancer patients for this study represent our target group. The response rate (65.7%), on the other hand, could influence the generalizability of our findings. The generalizability will also be influenced by the 170 respondents that were excluded from the study as they did not answer all the three questions concerning CAM. This might have led to an overestimated CAM use as respondents with missing answers might have been more likely to not have used CAM [28]. These patients did, on the other hand, not differ significantly from the informants answering all three CAM questions concerning gender, age, or income. 

The 12-month recall period concerning CAM use might likewise result in inaccuracies with regard to use. This factor should be equally distributed among women and men.

One of the three CAM questions asked for the use of herbal or “natural” medicine without defining this further. This could constitute an over- or underreporting of such use depending on how each participant defined their use and could also be differential between gender as men and women might define this in a different way.

It is also important to be aware of the fact that 37.8% of the women had breast cancer and 34.8% of the men had prostate cancer. One could, therefore, think that the gender-specific associations were connected to these cancer sites rather than gender itself, but this is shown to be unlikely as separate analyses excluding these two cancer sites were conducted with the same results, however, no longer at a statistical significant level. 

### 4.2. Prevalence

Many studies report the use of CAM in cancer patients, but the studied population, time frame in use, and definition of CAM varies widely. We have, therefore, chosen to compare our study to a limited selection of other studies with focus on comparability. 

A former Tromsø study conducted in 2001/2002, the fifth Tromsø study, found lower use of a CAM provider in the last 12 months than what we found six years later, 10.6% in women and 3.8% in men [29]. The reason for this is likely to be the strict legislation that regulated the CAM field at the time of the fifth study; only physicians and dentists were allowed to treat cancer patients. When the sixth Tromsø study was conducted in 2007/2008, this legislation had been considerably moderated. Also the preprepared list exemplifying CAM providers in the sixth study might have increased reported CAM use as this might have improved the recall and clarified what to consider as CAM. 

A Norwegian study, reporting CAM use in cancer patients with a poor survival prognosis at the time of first diagnosis, found that 22.7% had seen a CAM provider at least once after first diagnosis [28], 30% of the women and 14% of the men [23]. The reported use increased to 38.8% [28], 46% among women and 30% among men [23], when CAM techniques and OTC products were included. The somewhat higher use in that study might be due to the longer time frame of use (since diagnosis, at least 5 years) and the poorer prognosis in the studied population. 

Cancer patients in the county of Nord-Trøndelag, Central Norway were found to use a CAM provider to a larger degree than found in our study [19]. They found, contrary to us, that cancer patients were more likely to have seen a CAM provider than the total population. The difference in use might be due to a wider definition of a CAM provider in their study. Mao et al. found that 40% of all cancer survivors in a national sample in the US had used CAM within the last 12 months. They also found, contrary to us, that the cancer survivors were more frequent users than the total population. They found 45% CAM use in women and 33% CAM use in men [30]. The somewhat higher prevalence of use in their study might be due to their wider definition of CAM [23]. Average CAM use of 40% was also found in a systematic review presenting data from 152 studies in 18 countries representing more than 65 000 cancer patients. When limited to Europe, 34% CAM use was found which is very close to our findings. This study did not, however, provide gender-specific prevalence of use [31].

A large community-based national registry study in USA found that 33% of men with prostate cancer had used some sort of CAM. This is somewhat higher than what we found in men with cancer in our study and might be due to the specific cancer site. The US study also had a wider definition of CAM than what we had, but limited, on the other hand, the use to the last 6 months compared to our 12 months [32]. The use of CAM in Canadian men with prostate cancer was found to be 29.8% and was closer to our findings [33].

Our findings of CAM use in women were somewhat lower than what was found in recent studies in Europe, USA, and Australia [3, 17, 34–38], though some studies also found less use of CAM than what we found [39–41]. When less use was found, the CAM use was limited to a CAM provider [42] or to a newly diagnosed breast cancer patients [40]. The wide range of 16.5% to 87.9% reported use is partly due to the different ways of collecting data on CAM use (open questions and preprepared lists, different time frame of use, and current use to life time use) and different levels of use (level 2 to 6 in the NAFKAM model [23]). There were also differences with regard to the populations studied, varying from newly diagnosed breast cancer patients undergoing conventional treatment to national samples of women diagnosed with cancer. When these factors were taken into consideration, we still found a somewhat higher proportion of CAM users in most studies, especially American, Canadian, and Australian studies. This might be due to a more established tradition with integrated complementary cancer care compared to Norway and that most of these studies reported use in breast cancer patients only. 

The proportion of cancer patients using CAM in this study does not differ much from what was found in other studies when the comparison is restricted to comparable parameters. This shows how important it is to ensure comparability when studies are compared [23, 43]. It is important to define clearly with examples how to define a CAM provider and to use a standard questionnaire like the I-CAM-Q [44]. It is also important to clarify which level of CAM use was investigated and to report CAM use at more than one level as discussed in the NAFKAM cumulative model of reporting CAM use [23].

### 4.3. Associations

Our findings of CAM use associated with female gender and breast cancer are in accordance with findings in other studies [10, 45–48]. The reasons for higher CAM use in women might be explained by the fact that women use health services in general to a larger degree than men [49]. The increased use in breast cancer patients might be due to a high number of survivors suffering from severe side effects from conventional treatment and a somewhat younger cancer population more likely to feel their cancer as a threat to future plans [10] and care for children.

Different associations for CAM use in men and women concerning age and university education were also found in a recent Norwegian study [19]. Many find like us that female CAM users are more likely to have university education than nonusers. Women with university education might be more aware of CAM and more able to find relevant information about CAM. Young age [42, 50–52] and higher income [50, 51] have often been associated with CAM use in women. This was also found in our study, however not at a significant level. The reason for this might be that we have a strong tradition for the use of traditional healers among the elderly in Northern Norway and that these healers are classified as CAM providers in this study.

Our finding of lower income in male CAM users compared to nonusers is not in accordance with findings in other studies [33, 53–56]. The reason for this might be due to that more CAM users than nonusers have reached the age of 67 and as a consequence of this are likely to be retired from work. 

We found no association between education and CAM use in men. This is in accordance with several other studies [33, 56, 57]. Boon suggests that CAM use is no longer a phenomenon restricted to a unique segment of the population that is highly educated and enjoys a high family income [33]. This seems valid for our male CAM users. The lack of differences in educational level is not in accordance with what we found in women using CAM. The discrepancy between men and women in our study might be due to a general higher educational level among men. 

The tendency towards older age in overall CAM use and OTC products in men in our study is not found in other studies that we are aware of. Some studies found no associations between age and CAM use [57, 58], other found male CAM users to be younger than the nonusers [54, 56]. Inclusion of both traditional healers commonly used by elderly people and modern CAM providers used by the younger generations might explain the lack of significant age differences in our study. 

The findings of different associations for CAM use in men and women are important both for researchers and in clinical practice as the general impression of CAM users seems to be based on studies where the CAM users are dominated by women. This could give an incorrect impression of male CAM users. 

## 5. Conclusion

According to this study, prevalence and associations (age, education, and income) for CAM use differ significantly between male and female survivors of cancer. This underlines the importance of gender-specific analyses in future research.

## Figures and Tables

**Table 1:** Basic characteristics of the studied participants.

	Cancer patients	Women with cancer	Men with cancer
	(*n* = 630)	(*n* = 325)	(*n* = 305)
Percentage women	51.6		
Mean age	65.9	66.5	66.3
Median age (range)	66 (30–87)	66 (30–87)	67 (36–86)
Living with a spouse/partner %	70.2	56.8	84.4
University degree %	32.6	28.3	37.2
Self-reported good health %	53.0	53.1	53.0
Self-reported poor health %	9.0	8.4	9.6
More than 400 000 NOK (70 000$/54 000€) in house hold income last year %	47.5	38.9	55.8
Less than 125 000 NOK (21 500$/16 400€) in house hold income last year %.	3.5	5.7	1.4
Seen a general practitioner last year %	89.6	90.7	88.4
Mean time since diagnosis (years)	10.6	12.0	9.4

**Table 2:** Gender-specific CAM use in the last 12 months.

	Total	Women	Men	
	(*n* = 630)	(*n* = 325)	(*n* = 305)	*P* value
	%	%	%	
Have you during the last 12 months seen an alternative provider (homeopath, acupuncturist, foot zone therapist, herbal medicine practitioner, laying on of hands practitioner, healer, clairvoyant, etc.)?	(*n* = 79) 12.5	(*n* = 51) 15.7	(*n* = 28) 9.2	0.01
In the last 12 months have you used herbal or “natural” medicine?	(*n* = 155) 24.6	(*n* = 93) 28.6	(*n* = 62) 20.3	0.02
In the last 12 months have you used meditation, yoga, qi gong, or Tai Chi as a self-treatment?	(*n* = 29) 4.6	(*n* = 23) 7.1	(*n* = 6) 2.0	<0.01

Over all CAM use	(*n* = 213) 33.8	(*n* = 128) 39.4	(*n* = 85) 27.9	<0.01

**Table 3:** Overall CAM use. Socio demographic characteristics of users and nonusers.

	CAM users		Nonusers of CAM			CAM users		Nonusers of CAM			Interaction
	Women		Women		*P* value	Men		Men		*P* value	women/men
	(*n* = 128*)		(*n* = 197*)			(*n* = 85*)		(*n* = 220*)			
	(*n*)	%	(*n*)	%		(*n*)	%	(*n*)	%		*P* value
Age											
30–66 years	(75)	58.6	(102)	51.8	0.228	(32)	37.6	(108)	49.1	0.072	**0.032**
67–87 years	(53)	41.4	(95)	48.2		(53)	62.4	(112)	50.9		
Education											
Primary/secondary school	(80)	63.5	(148)	77.1	0.008	(54)	64.3	(135)	62.2	0.738	**0.041**
University education	(46)	36.5	(44)	22.9		(30)	35.7	(82)	37.8		
Family income											
Low to medium	(61)	47.7	(110)	55.8	0.149	(45)	52.9	(83)	37.7	0.016	**0.006**
Medium to high	(67)	52.3	(87)	44.2		(40)	47.1	(137)	62.3		
Living with a spouse/partner											
Yes	(74)	61.2	(106)	54.1	0.217	(69)	82.1	(185)	85.3	0.505	**0.211**
No	(47)	38.8	(90)	45.9		(15)	17.9	(32)	14.7		

*Due to missing response on one or more variables, the analysed numbers do not always add up to the total number.

**Table 4:** Overall CAM use. Health-related characteristics of users and nonusers.

	CAM users		Nonusers of CAM			CAM users		Nonusers of CAM			Interaction
	Women		Women		*P*-value	Men		Men		*P*-value	women/men
	(*n* = 128*)		(*n* = 197*)			(*n* = 85*)		(*n* = 220*)			
	(*n*)	%	(*n*)	%		(*n*)	%	(*n*)	%		*P*-value
Self-reported health											
Medium to good health	(114)	90.5	(181)	92.3	0.554	(74)	88.1	(199)	91.3	0.399	**0.852**
Poor health	(12)	9.5	(15)	7.7		(10)	11.9	(19)	8.7		
Time since diagnosis											
Less than one year	(5)	3.9	(5)	2.5	0.819	(3)	3.5	(17)	7.7	0.479	**0.737**
1–5 years	(33)	25.8	(46)	23.4		(34)	40	(74)	33.6		
5–10 years	(35)	27.3	(60)	30.5		(22)	25.9	(56)	25.5		
More than 10 years	(55)	43	(86)	43.7		(26)	30.6	(73)	33.2		
Cancer localization											
Breast	(57)	44.5	(66)	33.5	0.193						
Cervix uteri	(4)	3.1	(13)	6.6							
Other parts of uterus	(4)	3.1	(14)	7.1							
Ovary	(10)	7.8	(7)	3.6							
Prostate						(35)	41.2	(71)	32.3	0.439	
Testis						(3)	3.5	(17)	7.7		
Colon	(9)	7.0	(14)	7.1		(4)	4.7	(18)	8.2		
Bladder	(2)	1.6	(8)	4.1		(7)	8.2	(15)	6.8		
Rectum and anus	(4)	3.1	(4)	2.0		(5)	5.9	(10)	4.5		
Trachea, bronchus, and lung	(1)	0.8	(4)	2.0		(4)	4.7	(6)	2.7		
Lymphoid	(7)	5.5	(9)	4.6		(4)	4.7	(17)	7.7		
Kidney	(0)	0	(1)	0.5		(1)	1.2	(10)	4.5		
All other cancer sites	(30)	23.4	(57)	28.9		(22)	25.9	(56)	25.5		
Breast cancer											
Breast	(57)	44.5	(66)	33.5	0.045						
Other sites	(71)	55.5	(131)	66.5		(85)	100	(220)	100		
Prostate cancer											
Prostate						(35)	41.2	(71)	32.3	0.143	
Other sites	(128)	100	(197)	100		(50)	58.8	(149)	67.7		
Metastases											
Metastases at first diagnosis	(27)	21.1	(54)	27.4	0.415	(12)	14.1	(37)	16.8	0.487	**0.953**
No metastases	(73)	57	(106)	53.8		(49)	57.6	(110)	50		
Unknown	(28)	21.9	(37)	18.8		(24)	28.2	(73)	33.2		

*Due to missing response on one or more variables, the analysed numbers do not always add up to the total number.

**Table 5:** CAM provider. Basic characteristics of users and nonusers.

	CAM provider		No CAM provider			CAM provider		No CAM provider			Interaction
	Women		Women		*P*-value	Men		Men		*P*-value	women/men
	(*n* = 51*)		(*n* = 274*)			(*n* = 28*)		(*n* = 273*)			
	(*n*)	%	(*n*)	%		(*n*)	%	(*n*)	%		*P*-value
Age											
30–66 years	(28)	54.9	(149)	54.4	0.945	(12)	42.9	(128)	46.2	0.734	0.757
67–87 years	(23)	45.1	(125)	45.6		(16)	57.1	(149)	53.8		
Education											
Primary/secondary school	(38)	76	(190)	70.9	0.462	(16)	57.1	(173)	63.4	0.516	0.335
University education	(12)	24	(78)	29.1		(12)	42.9	(100)	36.6		
Family income											
Low to medium	(27)	52.9	(144)	52.6	0.960	(14)	50	(114)	41.2	0.366	0.497
Medium to high	(24)	47.1	(130)	47.4		(14)	50	(163)	58.8		

*Due to missing response on one or more variables, the analysed numbers do not always add up to the total number.

**Table 6:** OTC products. Basic characteristics of users and nonusers.

	OTC products		No OTC products			OTC products		No OTC products			Interaction
	Women		Women		*P*-value	Men		Men		*P*-value	women/men
	(*n* = 93*)		(*n* = 232*)			(*n* = 62*)		(*n* = 243*)			
	(*n*)	%	(*n*)	%		(*n*)	%	(*n*)	%		*P*-value
Age											
30–66 years	(55)	59.1	(122)	52.6	0.284	(22)	35.5	(118)	48.6	0.065	0.036
67–87 years	(38)	40.9	(110)	47.4		(40)	64.5	(125)	51.4		
Education											
Primary/secondary school	(56)	61.5	(172)	75.8	0.011	(42)	68.9	(147)	61.3	0.273	0.014
University education	(35)	38.5	(55)	24.2		(19)	31.1	(93)	38.8		
Family income											
Low to medium	(47)	50.5	(124)	53.4	0.635	(32)	51.9	(96)	39.5	0.085	0.108
Medium to high	(46)	49.5	(108)	46.6		(30)	48.4	(147)	60.5		

*Due to missing response on one or more variables, the analysed numbers do not always add up to the total number.

**Table 7:** CAM techniques. Basic characteristics of users and nonusers.

	CAM techniques		No CAM techniques			CAM techniques		No CAM techniques			Interaction
	Women		Women		P-value	Men		Men		P-value	women/men
	(*n* = 23*)		(*n* = 302*)			(*n* = 6*)		(*n* = 299*)			
	(n)	%	(n)	%		(n)	%	(n)	%		P-value
Age											
30–66 years	(20)	87	(157)	52	0.001	(4)	66.7	(136)	45.5	0.419	0.550
67–87 years	(3)	13	(145)	48		(2)	33.3	(163)	54.5		
Education											
Primary/secondary school	(10)	43.5	(218)	73.9	0.002	(2)	33.3	(187)	63.4	0.200	0.987
University education	(13)	56.5	(77)	26.1		(4)	66.7	(108)	36.6		
Family income											
Low to medium	(9)	39.1	(162)	53.6	0.179	(1)	16.7	(127)	42.5	0.407	0.625
Medium to high	(14)	60.9	(140)	46.4		(5)	83.3	(172)	57.5		

*Due to missing response on one or more variables, the analysed numbers do not always add up to the total number.
